# Supplementary material for: Evaluating the implementation of weekly rifapentine-isoniazid (3HP) for tuberculosis prevention among people living with HIV in Uganda: A qualitative evaluation of the 3HP Options Trial
Source: PLOS Glob Public Health. 2024 Oct 24;4(10):e0003347. doi: 10.1371/journal.pgph.0003347 (PMC11500930; doi:10.1371/journal.pgph.0003347)
Supplement: S1 Checklist — (DOCX) [file pgph.0003347.s001.docx]

**CONSOLIDATED CRITERIA FOR REPORTING QUALITATIVE RESEARCH (COREQ): 32-ITEM CHECKLIST**

| **No. item** | **Guide questions/description** | **Reported on page no.** | **How the manuscript adheres to the reporting standards** |
| --- | --- | --- | --- |
| **Domain 1: Research team and reﬂexivity** | | | |
| *Personal Characteristics* | | | |
| 1. Interviewer/facilitator | Which author/s conducted the interview or focus group? | 9 | We describe who led the qualitative research team and who conducted the interviews including their credentials, occupation, gender, and experience/ training in qualitative research. |
| 2. Credentials | What were the researcher’s credentials? E.g. PhD, MD | 9-10 |  |
| 3. Occupation | What was their occupation at the time of the study? | 9-10 |  |
| 4. Gender | Was the researcher male or female? | 9-10 |  |
| 5. Experience and training | What experience or training did the researcher have? | 9-10 |  |
| *Relationship with participants* | | | |
| 6. Relationship established | Was a relationship established prior to study commencement? | 9 | We report that the interviewer did not know the study participants prior to study commencement.  We report that the interviewer established rapport with all participants and shared the objectives of the interview before starting the interview.  We do not describe interviewer characteristics like bias, assumption, and others. |
| 7. Participant knowledge of the interviewer | What did the participants know about the researcher? e.g. personal goals, reasons for doing the research | 9 |  |
| 8. Interviewer characteristics | What characteristics were reported about the interviewer/facilitator? e.g. Bias, assumptions, reasons and interests in the research topic | N/A |  |
| **Domain 2: study design** | | | |
| *Theoretical framework* | | | |
| 9. Methodological orientation and Theory | What methodological orientation was stated to underpin the study? e.g. grounded theory, discourse analysis, ethnography, phenomenology, content analysis | 6, 10, 11 | We describe the use of the RE-AIM implementation science framework and inductive/deductive thematic analysis. |
| *Participant selection* | | | |
| 10. Sampling | How were participants selected? e.g. purposive, convenience, consecutive, snowball | 8 | We describe the purposive sampling technique we used to select participants, how we approached the participants, and the sample size.  We report that participants were approached in person during clinic visits and via telephone during the COVID-19 pandemic.  None of the participants refused to participate in the interviews. |
| 11. Method of approach | How were participants approached? e.g. face-to-face, telephone, mail, email | 8-9 |  |
| 12. Sample size | How many participants were in the study? | 9 |  |
| 13. Non-participation | How many people refused to participate or dropped out? Reasons? | N/A |  |
| *Setting* | | | |
| 14. Setting of data collection | Where was the data collected? e.g. home, clinic, workplace | 9 | We describe where and how interviews were conducted.  We report that only the interviewer and participant were present during the interviews.  We state the dates of data collection and describe the demographic characteristics of the participants in the study results. |
| 15. Presence of non-participants | Was anyone else present besides the participants and researchers? | 9 |  |
| 16. Description of sample | What are the important characteristics of the sample? e.g. demographic data, date | 9, 11 |  |
| *Data collection* | | | |
| 17. Interview guide | Were questions, prompts, guides provided by the authors? Was it pilot tested? | 9, 10 | We describe the PLHIV and healthcare provider interview guides, the interview topics, and how the interview guides were piloted.  There were no repeat interviews.  We report that all interviews were audio-recorded. We report that interviews lasted 35-50 minutes. We describe how we arrived at data saturation.  Transcripts were not returned to participants for comment or correction. |
| 18. Repeat interviews | Were repeat interviews carried out? If yes, how many? | N/A |  |
| 19. Audio/visual recording | Did the research use audio or visual recording to collect the data? | 10 |  |
| 20. Field notes | Were ﬁeld notes made during and/or after the interview or focus group? | N/A |  |
| 21. Duration | What was the duration of the inter views or focus group? | 9 |  |
| 22. Data saturation | Was data saturation discussed? | 9-10 |  |
| 23. Transcripts returned | Were transcripts returned to participants for comment and/or correction? | N/A |  |
| **Domain 3: analysis and ﬁndings** | | | |
| *Data analysis* | | | |
| 24. Number of data coders | How many data coders coded the data? | 10 | We describe how data was coded using NVivo by two researchers, the debrief process with two other members of the study team, and how the coding framework was developed.  We describe how themes were derived from the data using an inductive thematic approach and deductive alignment of themes to the dimensions of the RE-AIM framework.  Participants provided feedback on the findings. |
| 25. Description of the coding tree | Did authors provide a description of the coding tree? | 10 |  |
| 26. Derivation of themes | Were themes identiﬁed in advance or derived from the data? | 10-11 |  |
| 27. Software | What software, if applicable, was used to manage the data? | 10 |  |
| 28. Participant checking | Did participants provide feedback on the ﬁndings? | 11 |  |
| *Reporting* | | | |
| 29. Quotations presented | Were participant quotations presented to illustrate the themes/ﬁndings? Was each quotation identiﬁed? e.g. participant number | 12-24 | Quotations with clear identification are presented to illustrate the analytical findings. We believe that the results are clearly presented and well-discussed. |
| 30. Data and ﬁndings consistent | Was there consistency between the data presented and the ﬁndings? | 12-24 |  |
| 31. Clarity of major themes | Were major themes clearly presented in the ﬁndings? | 25-27 |  |
| 32. Clarity of minor themes | Is there a description of diverse cases or discussion of minor themes? | 25-27 |  |
